# Elliptic Curve Cryptography Considerations for Securing Automation and SCADA Systems

**DOI:** 10.3390/s23052686

**Published:** 2023-03-01

**Authors:** Alexandra Tidrea, Adrian Korodi, Ioan Silea

**Affiliations:** Department of Automation and Applied Informatics, Faculty of Automation and Computers, University Politehnica Timișoara, 300223 Timișoara, Romania

**Keywords:** automation systems, ECC, ECDSA, ECIES, OPTIGA TrustX, SCADA security

## Abstract

Securing critical infrastructures and manufacturing plants in the Industrial-Internet-Of-Things and Industry 4.0 is a challenge today due to the increased number of attacks against automation and SCADA systems. These systems were built without any security considerations in mind, so the evolution towards interconnection and interoperability makes them vulnerable in the context of exposing data to the outside network. Even though new protocols are considering built-in security, the widely spread legacy standard protocols need to be protected. Hence, this paper attempts to offer a solution for securing the legacy insecure communication protocols based on elliptic curve cryptography while fulfilling the time constraints of a real SCADA network. Elliptic curve cryptography is chosen to respond to the low memory resources available for the low level devices of a SCADA network (e.g., PLCs), and also because it can achieve the same level of security as other cryptographic algorithms using smaller sizes for the secure keys. Furthermore, the proposed security methods have the purpose of assuring that the data transmitted between entities of a SCADA and automation system are authentic and confidential. The experimental results showed good timing performance for the cryptographic operations executed on Industruino and MDUINO PLCs, demonstrating that our proposed concept is deployable for Modbus TCP communication in a real automation/SCADA network on existing devices from the industry.

## 1. Introduction

SCADA (Supervisory Control and Data Acquisition) and automation systems are in charge of controlling vital infrastructures for the well-functioning of our society, such as health systems, transportation systems, water, energy, economy and national security. A cyber-attack conducted on critical infrastructures can lead to the disruption of normal processes and even physical damage, having a major impact on the end users of the provided services. Stuxnet [1], the Florida water treatment plant attack [2], Ukraine power outage [3] and Colonial Pipeline ransomware [4] are only some examples of cyber-attacks which have raised awareness among governments and researchers, while they also emphasize the urgent need for protecting and securing critical infrastructure. Starting with Industry 4.0, which brings the need for assuring interoperability and interconnectivity, an air-gap network is no longer considered as efficient in securing infrastructures [5]. Even though in the context of Industrial-Internet-Of-Things (IIoT) and cyber-physical systems (CPS) new systems and protocols are deployed, the legacy automation and SCADA systems are widely used in plants despite their lack of security mechanisms due to legacy structures.

Automation and SCADA systems are evolving towards Industry 4.0 and IIoT concepts. Legacy systems and solutions have received the most coverage. Currently, at hierarchical levels up to the PLC, legacy protocols are implemented with a prominent usage of Modbus, and, when Ethernet is present, Modbus TCP. Even new PLC equipment has to cope with smarter measuring devices and other legacy solutions that have only Modbus, S7, etc. Horizontally, the PLC level in the industry is communicated through Modbus TCP, Profinet, Ethernet/IP, etc., and yet only a few are using OPC UA. PLC-SCADA communication follows mostly legacy patterns, either legacy Ethernet-based protocols, or centralized OPC UA servers converting the same protocols on the lower side. Although the industrial tendency is to use OPC UA, where specifications including security are being continuously researched, the industry has to secure legacy protocols-based communications. Currently, with the lack of available and proper security solutions at the protocol level, the industry is struggling to achieve important Industry 4.0 objectives.

In the abovementioned context, the authors propose an approach for legacy communication protocols to secure the transferred data between lower levels of an industrial system architecture based on elliptic curve cryptographic methods and an Optiga Trust X security controller. The proposed concept is highlighted for the widely spread Modbus TCP legacy communication protocol, and the entire solution is developed and evaluated on devices used in industry for automation and SCADA systems. In addition, our contribution is emphasized by the technological readiness level and by the possibility of deploying the proposed concept, which provides the means for assuring authenticity and confidentiality on legacy communication protocols other than Modbus TCP.

The major contributions of this paper can be summarized as follows:

1. We design and implement a solution for securing Modbus TCP communication within automation and SCADA systems based on the Elliptic Curve Digital Signature Algorithm (ECDSA) and Elliptic Curve Integrated Encryption Scheme (ECIES) as elliptic curve cryptographic methods, and use Optiga Trust X as a security controller.
2. We implement our security solution using open-source C language libraries, which were adapted and optimized to fit low memory resources and timing constraints of a real SCADA network. In addition, we built and configured a cryptographic library to allow the integration of various elliptic curves.
3. We integrate within our solution an open-source software library for Optiga Trust X to perform ECC key generation and execution of cryptographic operations corresponding to an authentication cryptographic scheme based on ECDSA.
4. We integrate and deploy our approach on Industruino PLCs and MDUINO PLC, devices used in industry. Moreover, we integrate the security chip Optiga Trust X within the experimental setup as an additional element for providing secure key storage and fast execution of the cryptographic operations.
5. We measure, in the experimental setup, the duration of each cryptographic operation and evaluate for each cryptographic method the security level introduced and the latency added by our approach. In addition, we perform a security analysis which addresses the security protection introduced by the proposed concept against MITM and replay attacks.

The paper describes the relevant literature studies for the subject of this paper in Section 2. Section 3 contains a description of the proposed concept from design to implementation. The following section has the purpose of describing the experimental setup from hardware and software perspectives. Section 5 contains the experimental results, their interpretation and the discussions related to the obtained results. In Section 6, the conclusions of our work and the benefits for securing industrial systems are presented.

## 2. Related Work

The literature addresses critical infrastructure security from various perspectives, starting with the analysis of vulnerabilities of legacy structures towards providing security guidelines to be applied at different levels of architecture up to attempts of providing solutions for specific issues raised as a result of exposing the SCADA and automation systems to the outside world in the context of evolution towards IIoT. In this context, a study by Embed Security [6] showed different security vulnerabilities if SCADA monitoring and control applications are exposed through mobile applications, such as insecure authorization, the improper implementation of authentication methods, insecure channel communication (weak handshake, exposure of sensitive assets) and inefficiently applied cryptography (hard-coded encryption keys, fixed initialization vectors).

With respect to vulnerabilities, SCADA and automation systems are liable to attacks on communication protocols, hardware devices and software components. One important aspect to be considered when analyzing vulnerabilities of SCADA and automation systems is related to PLC (programmable logic controller) security, and more specifically, to payload and firmware attacks [7]. Attackers can thus gain access through compromising software applications and change the execution mode of the process, which can lead to physical damage. Moreover, vulnerabilities in the authentication protocols used by several PLC vendors were addressed by [8], while [9] exploited vulnerabilities of PLCs as part of a smart city network.

The authors in [10] emphasize the lack of cybersecurity mechanisms for assuring authenticity, integrity and confidentiality for the classical network communication protocols; vulnerabilities which can be exploited by an intruder in order to conduct denial-of-service (DoS), man-in-the-middle (MITM), message spoofing, replay or data injection attacks. In [11], a lack of task prioritization, database injection and issues at the communication protocol level were identified as major threats to SCADA systems. Additionally, emphasized a lack of integrity checks and anti-replay mechanisms among common vulnerabilities for several industrial communication protocols [12], such as Profibus, DNP3 and Modbus.

Furthermore, the authors from [13] highlighted buffer errors, authentication issues and insufficient cryptography as vulnerabilities at different architectural levels of a SCADA system identifying, as a major attack vector, the network with 89% exploitable assets. Cross-site scripting and SQL injection were added to the attack vector list for SCADA systems by [14], which identified a major concern in the high usage in the field of legacy communication protocols with Modbus as the most frequently used, even though it was evaluated as insecure by [15]. Since the legacy communication protocols were built without having the need for protection against cyberattacks in mind, new protocols with built-in security mechanisms have been developed, as stated by [16], with a focus on OPC UA [17]. Nevertheless, even though the legacy communication protocols do not fulfill the cybersecurity requirements imposed by the current standards, it is a big challenge to completely replace them due to a high number of interconnected devices. Therefore, methods for introducing security mechanisms or enhancing existing ones are required.

### 2.1. Security of SCADA and Automation Systems in Industry 4.0

Securing SCADA and automation systems is addressed by the literature by proposing various methods to mitigate the identified vulnerabilities. Worth mentioning are the challenges which are introduced by Industry 4.0, such as providing real-time data access, methods to allow the integration of a high amounts of sensors and devices in a secure manner and live tools for error identification [18]. With respect to the proposed concept of this paper, which addresses issues related to insecure communication and low memory resources, the authors mention the relevant ones. The literature addresses the vulnerabilities of classical communication protocols, such as Modbus and DNP3, by focusing on providing solutions for enhancing their security, or by proposing intrusion detection mechanisms. One of the problems consists of a lack of efficient authentication methods, addressed by [19], where a one-way authentication method based on a hash function was proposed, which was demonstrated by [20] as being vulnerable to attacks leading to cases where the master was impersonated or the slave could no longer authenticate. A solution for providing mutual authentication for devices communicating on the Modbus TCP protocol was proposed by [21], based on the stream control transmission protocol (SCTP) and authentication messages based on a hash-based message authentication code (HMAC), but without addressing the secure storage of the cryptographic keys, which is a common issue of all security solutions only implemented through software. In order to respond to the lack of suitable integrity and authenticity methods, in 2008, a secure version of Modbus TCP [22] was published, which used TLS as packet encapsulations. A solution was proposed in [23] for encrypting communication on Modbus TCP based on SSL/TLS with the chacha20-poly1305 encryption algorithm tested on a PC. The paper [24] presented a solution for encrypting the communication using TLS 1.3 and authentication between a control server and a switch. A trusted server was used for authentication, which generated tokens for lower level devices. However, none of the TLS-based approaches included detailed analysis conducted by researchers related to the latency introduced by the proposed security mechanisms.

With respect to intrusion detection mechanisms, the approach presented by [25] collected attacks through a honeypot called Conpot. The method has been shown to demonstrate high accuracy in identifying attack patterns based on the tests deployed on a smart meter. The study in [26] proposed a method for detecting DoS attacks, while [27] proposed a decentralized method for intrusion detection of attacks focused on compromising the integrity of transmitted data.

Another direction in research in respect to the IIoT concept is the addition of new security mechanisms in critical infrastructure architecture, such as blockchains [28,29], digital signatures [30] or quantum cryptography [31]. In this case, only software-based solutions are proposed. However, the literature proposes hardware- and software-based solutions in order to ensure hardware security alongside software security, such as using trusted platform modules (TPM) [32,33] or hardware devices as part of a SCADA network for authentication mechanisms [34]. The TPMs can be used for secure key storage, protecting against key cloning and key extraction physical attacks.

### 2.2. Elliptic Curve Cryptography Solutions

Elliptic curve cryptography (ECC) is an alternative to classical cryptographic mechanisms, such as RSA, since it offers the same security level for smaller key sizes, as shown in [35], and it can be utilized for secret key generation and exchange schemes, encryption and decryption of sensitive data or in the context of digital signatures. Furthermore, the ECC-based protocols have a higher energy efficiency than RSA, as shown in [36]. Regarding the security mechanisms of the secret keys, presented a solution for key management within a SCADA network based on elliptic-curve Diffie–Hellman (ECDH) [37], which had the purpose of increasing security for the key of the master device. However, its applicability on a real SCADA network and the latency of the proposed solution was not analyzed. A trusted ID-based solution for establishing secure session keys alongside a digital signature algorithm was proposed in [38] for securing communication between PLCs and intelligent electronic devices of a SCADA system. In this case, only a formal security analysis was conducted for the proposed solution. In the same direction, presented a scheme for a secure key exchange based on NTRU public key cryptosystems [39], a Vernon cypher for key generation and HMAC for message authenticity. Nevertheless, the proposed architecture was not evaluated on hardware devices within a SCADA network, and solutions for secure storage of the secret keys within the new structure were not addressed.

With respect to digital signatures, authors from [40] proposed a solution for providing integrity protected communication within a wireless sensor network based on ECDSA, a security mechanism implemented by [41] to assure data integrity on a SCADA system interconnected to a cloud platform. Similar to this approach, the authors of [42] used ECDSA for the gateway authentication feature of a building automation system. Nevertheless, even though the Infineon Optiga Trust E, as an alternative to Infineon Optiga TPM, was highlighted as a solution for secure storage of the secret keys, the computational costs for the cryptographic functions were not presented. Furthermore, regarding the encryption and decryption of data using elliptic curve cryptography, there have been hybrid schemes which employ the generation of cryptographic keys using an ECC feed as input to symmetric encryption algorithms, as shown by [43], where the hybrid algorithm ECIES was analyzed against different standard requirements. In [44], ECIES was used for encrypting data transmitted by the IoT devices and hash algorithms for signatures, while [45] provided a mutual RFID-based authentication between a server and a tag of a IoT device using ECC. Even though security analysis of the proposed algorithm using AVISPA was carried out, the latency and computational costs introduced by each cryptographic operation were not evaluated.

In summary, to the best of our knowledge, our work is among the first studies to provide a solution based on ECC and Optiga Trust X for securing legacy communication protocols, such as Modbus TCP, while complying with the time constraints and low memory resources requirements of SCADA and automation systems. To our knowledge, only two research papers have approached ECC integration with Modbus TCP. The first paper [46], our own previous work, integrated elliptic curve digital signatures as a security mechanism to protect against MITM attacks with the help of a trusted platform module, but without being deployed on devices from the industry. The second paper [47], only focused on integrating the Elliptic-curve Diffie-Hellman algorithm as a method for secure key exchange and the evaluation of introducing encryption functionality on Modbus TCP. However, the concept was deployed on a simulated environment and no security analysis was conducted in the work. As far as we could tell, there have been no studies conducted using our approach with respect to Modbus TCP secure communication, which are integrating and deploying ECIES on industrial devices. Moreover, we embedded Optiga Trust X as a security chip for secure key storage, ECC key generation and the execution of cryptographic operations for implementing ECDSA as an authentication method between devices within a SCADA network.

## 3. Proposed Concept

In order to address the security challenges posed by the requirements of IIoT, to assure the horizontal and vertical interoperability of systems, the authors approach has the goal of presenting a solution based on ECC for securing the network communication between devices from the lower levels of an operational technology architecture. Moreover, the intent of the paper is to demonstrate the integration of the proposed solution on equipment related to the industrial context through experiments. Concerning the approached legacy communication protocol, the authors chose Modbus TCP for implementing the proposed concept, since it has no security in place even though it is prevalent in industry. Nevertheless, the proposed concept can be extended to other legacy communication protocols, such as DNP3, S7, etc. Securing legacy protocols-based communication and deploying security mechanisms on devices with low memory resources and low computational costs while allowing real-time data access inside PLC-SCADA communication are the major security challenges addressed by the proposed concept in the context of achieving Industry 4.0 objectives. Furthermore, our work aims to address MITM, cyphertext attacks and replay attacks by providing a solution to ensure the authenticity, integrity and confidentiality of exchanged messages within an automation and SCADA network.

**Figure 1:**
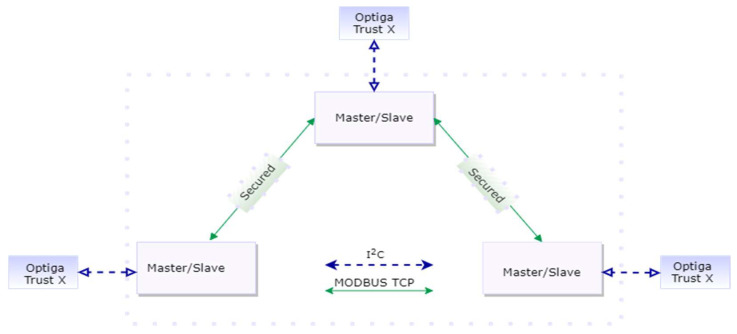
System Architecture.

As described in Figure 1, the system architecture consists of independent system elements, which can play the master or slave role during a communication over Modbus TCP within an automation/SCADA network. The authors propose two methods that can be considered independent or complementary in order to address needs for authenticity, integrity and confidentiality on insecure communication channels. One can argue that in order to communicate over a channel, two entities must have previously proven their identities in order to establish mutual trust. The first method addresses the aspect of being able to allow system elements to prove their identity before the communication occurs through digital certificates. In this way, the authenticity among system elements of the system of interest can be established. The second method responds to the need for assuring confidentiality and integrity by implementing a hybrid encryption scheme, which protects against adaptive cyphertext attacks while being able to handle large amounts of data at once with less computational costs than classical methods, as concluded by [48].

As an option to enhance the security of the proposed concept, for each system element, the authors implement an Optiga Trust X device in order to emphasize the advantages of using a security controller, such as secure key storage, and the fast execution of cryptographic operations. For each proposed method, the latency introduced by each cryptographic operation is evaluated along with the duration of each chosen elliptic curve.

### 3.1. Cryptographic Authentication Scheme

In order to provide a solution for data authenticity between a master and a slave within an automation/SCADA network, ECDSA is selected with a focus on the usability of a hardware security accelerator in terms of the concerns of the level of security introduced. As an authentication scheme, the ECDSA was chosen due to its smaller key sizes than other digital signatures, and its performance against cyber-attacks. The authors selected Optiga Trust X as a secure cryptographic microprocessor due to its capabilities in terms of secure key storage, a crypto engine, low computational costs and easy integration with low memory devices, since it has been optimized for IIoT. It also provides an efficient power management, which enables the possibility of configuring the power consumption of the chip in a specific range. Furthermore, this device has a lower acquisition cost than more complexes devices, such as trusted platform modules. A lower cost represents an advantage when analyzing the upgrading of a legacy SCADA and automation system with numerous devices. Worth mentioning is that the security module is designed as a turnkey, delivered with four ECC key slots from which the first one is assigned to the unique ECC key pair generated during production of the chip. The private key of the security module is stored in its built-in tamper-protected non-volatile memory (NVM), while the public key is used to generate an X.509 endorsement certificate which can be used to prove the authenticity of the chip. The remaining key slots can be used to securely store the private keys generated for performing cryptographic operations, such as for computing a digital signature and encryption or decryption operations. Therefore, having the private keys stored in the Optiga’s secured memory, an attacker will not be able to retrieve the private key and impersonate the issuer of the digital signature.

The Optiga Trust X offers the possibility to select NIST P-256 and NIST P-384 curves for generating the ECC key pair. For the proposed concept, the NIST P-256 was chosen with SHA-256 as a hash function for the ECDSA method. The authors did not address the method of interchanging the public key generated for each system element, but mention the requirements for having the necessary inputs available for the digital signature algorithm. Therefore, the following steps are required to be performed in a third-party secure environment for the first time when the system elements are connected:

- Step1: Generate the key pair (kDevicePrivECC, kDevicePubECC) using the security chip for each system element.
- Step2: Store kDevicePrivECC into the secure memory of the security chip.
- Step3: Load kDevicePubECC for each device into the security chip of the other master or slave device by performing an exchange of public keys, as follows:
  1. Load kDevicePubECC of the slave device in the security chip associated with the master device.
  2. Load kDevicePubECC of the master device in the security chip associated with the slave device.

The same steps are required to be performed for each system element that is an active participant in the communication over Modbus TCP in order to achieve mutual authentication inside the SCADA network. Nevertheless, the solutions for establishing trust for multiple participants may differ in respect to the architecture of the network and are not addressed in this paper.

**Figure 2:**
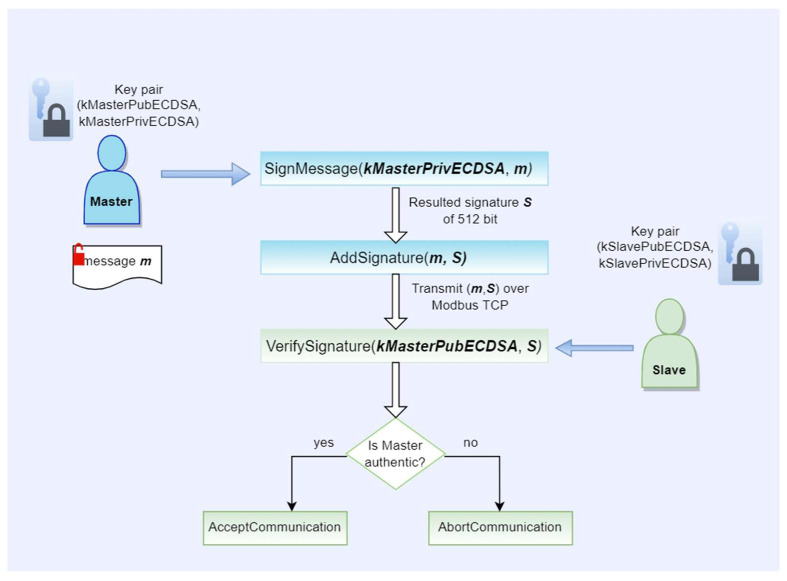
ECDSA authentication scheme.

With the key pairs generated and the public keys exchanged, at system startup, it is required that each master or slave device loads the public key of the other from the non-secure memory of its associated security chip. The principles of the cryptographic scheme are described in Figure 2, where the master acts as an initiator of the authentication and the slave plays the role of the verifier of the computed signature, which is executed as follows once each system is up and running:

- Step1: The master computes a hash over the message m using SHA256 primitive.
- Step2: The master signs the hashed message m with the secret key kMasterPrivECC computing a digital signature S of a 512-bit length.
- Step3: The obtained signature is added as an extension of the message m and sent over the Modbus TCP insecure channel to the slave device.
- Step4: The slave device receives the message m and the signature S in one frame and computes the hash over the message m.
- Step5: The resulted hash and the kMasterPubECC are used by the slave device to verify the computed signature.
- Step6: The result of the signature verification can lead to two options:
  1. If the verification is successful, the slave device accepts the request and further communication with the master device.
  2. If the signature is invalid, the slave device rejects the request and aborts the communication.

The same protocol is applicable when the slave initiates a request, or in case it sends a response by performing the same steps over the message in order to allow the master to verify the identity of the slave. In this way, the protocol allows bidirectional communication between the master and each slave, and can offer protection against MITM attacks. The ECDSA algorithm can be implemented and deployed on the system of interest without having an Optiga Trust X security chip having, as a base, the same principles for the cryptographic authentication scheme. However, since all the cryptographic operations will have to be executed by each device instead of the security chip, one must analyze the aspects related to the computational costs introduced and options for secure storage of the secret keys.

### 3.2. Integrated Encryption Scheme

The ECIES is a hybrid encryption scheme that uses ECC-based asymmetric cryptography and symmetric cyphers for providing encryption of the sensitive data in order to assure its confidentiality. Furthermore, the hybrid encryption scheme supports key generation, key derivation, key exchange mechanisms and message authentication. The integrated encryption scheme has various implementation methods, even though it was standardized by ANSI, IEEE, SECG and ISO/IEC. A comparison of the requirements and implementation guidelines detailed by each standard is presented in [49], with the conclusion that a generic solution for fulfilling all these standards when implementing the encryption scheme cannot be provided, as also stated by [50]. The integrated encryption scheme was chosen based on ECC performance on low computational devices with low memory resources, described in the next section, which fits the specifications of legacy devices such as PLCs used in the industry.

The proposed concept aims to prove the integration of ECIES on low level devices of a SCADA network in order to enhance their security and to offer a method for assuring confidentiality of data transmitted over an insecure channel. The cryptographic primitives and algorithms for performing the integrated encryption scheme were selected by the authors based on two reasons. The first is related to limited resources on the automation devices, while the second aims to satisfy the minimum security requirements in respect to known vulnerable attacks. For each operation, the duration and performance is evaluated. The relic toolkit cryptographic library offers the possibility of selecting several elliptic curves for key generation and it allows ECIES configuration. Therefore, the selected options and additional considerations for ECIES configuration based on the relic toolkit library is listed as follows, which is used for deploying the proposed concept:

- NIST P-160 and NIST P-192 curves are chosen for elliptic key pair generation
- SHA-256 is chosen as a hashing function
- AES-128 in CBC mode is used.

**Figure 3:**
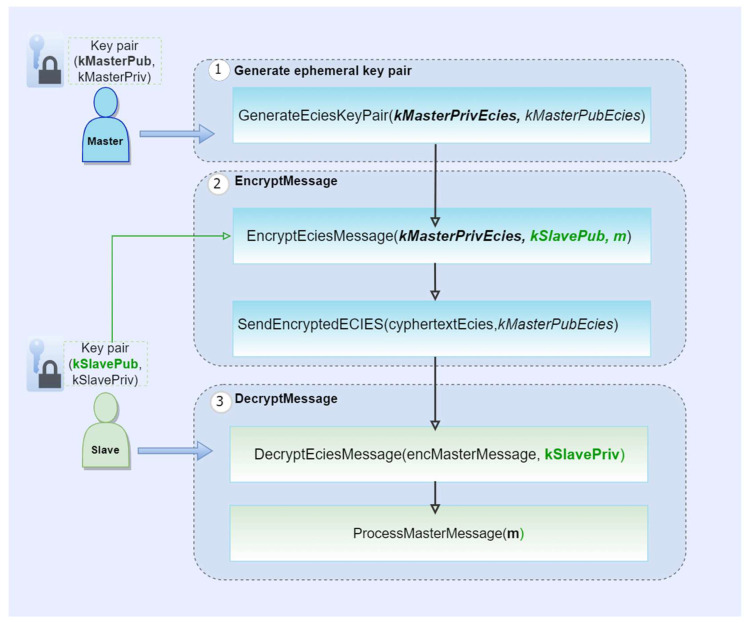
ECIES integrated encryption scheme.

Additionally, relic toolkit implementation of ECIES uses KDF2 as a key derivation function, HMAC-SHA256 as a message authentication code function and Diffie-Hellman (DH) as a key agreement function. In order to attain secure key management, the authors recommend that the generation is carried out in a secure environment by a third party for each device. Starting with the assumption that each device knows the others public key, and that this key is stored encrypted in the device’s memory or in a security chip, the steps of the proposed algorithm are shown in Figure 3 and detailed as follows:

- Step1: The master generates an ephemeral key pair (kMasterPrivECIESC, kMasterPubEcies) using the ECC-based selected elliptic curve.
- Step2: To encrypt the plaintext message m, the master device needs as input the ephemeral private key kMasterPrivECIESC, the slave’s public key kSlavePubECC. The encryption scheme contains several cryptographic operations which must be executed by the master device, as follows:
  1. Generate a shared secret s using DH algorithm based on the master ephemeral private key and slave public key.
  2. Generate the keys kEnc and kMac using KDF2 based on the shared secret s.
  3. Encrypt the message m using AES-128 in CBC mode having as input the symmetric key kEnc.
  4. Compute HMAC-SHA256 over the encrypted message c using the secret key kMac as input.
  5. Concatenate the encrypted message c with the resulted keyed hash h obtaining the output message cyphertextEcies.
  6. Send a message to the slave device composed of cyphertextEcies and master ephemeral public key kMasterPubEcies.
- Step3: To decrypt the received message cyphertextEcies and obtain the original message m, the slave device needs as input master ephemeral public key kMasterPubEcies and its private key kSlavePrivECC. The decryption scheme contains several cryptographic operations which must be executed by the slave device, as follows:
  1. Retrieve from cyphertextEcies the encrypted message c and the corresponding keyed hash h.
  2. Reproduce the shared secret s using DH algorithm based on the master ephemeral public key and slave private key.
  3. Generate a symmetric key kEnc and a secret key kMac using KDF2 based on the shared secret s.
  4. Compute HMAC-SHA256 over the received encrypted message c using the secret key kMac obtaining a keyed-hash h∗.
  5. Compare the resulted keyed-hash h∗ with the one received as input h. If they match the slave will perform further the decryption of the message. Otherwise, the slave will reject the message since there is a failure in message authentication code verification.
  6. Decrypt the message c using the AES-128 in CBC mode having as input the symmetric key kEnc.
  7. Process the original message m obtained after a successful decryption process.

Considering the same protocol, the bidirectional communication between a master and a slave can be encrypted using ECIES in order to respond to the need of protecting sensitive data, offering confidentiality and integrity. Even if an attacker intercepts the message, the original data transmitted cannot be recomputed without having access to the ECC keys used by the algorithm. To secure the communication channel between the two devices, first the authentication procedure must take place, followed by the integrated encryption procedure. In order to be resistant against replay attacks, a timestamp needs to be added to each transmitted message (must be known by the receiver or received by both parties) for both the authentication scheme and hybrid encryption scheme.

## 4. Experimental System Setup

In order to deploy our proposed concept on actual devices used in industry within a SCADA and automation system architecture, we chose two Industruino devices and one MDUINO device. The Industruino IND.I/O D21G and Industruino PROTO D21G are two controllers with capabilities for industrial and automation projects based on a ATSAMD21G microcontroller with 256 KB flash memory and a 32 KB SRAM. MDUINO PLC 21+ is used in industrial automation projects, such as for water treatment, being open-source hardware with ATmega2560 as a microcontroller, 256 KB flash memory and 8 KB SRAM. In terms of clock speed, the Industruino devices run at 48 Mhz, while the MDUINO device has a clock speed of 16 MHz. All three devices run open-source C language code, being fully compatible with ARDUINO IDE and having dedicated open-source libraries, which is an advantage from the perspectives of cost and customization possibilities. Additionally, they have integrated communication interfaces used in the industry, such as RS-232, I2C, RS-485, Ethernet and the Modbus protocol. In order to emulate an industrial communication network, we chose Modbus TCP as the communication protocol between the devices used in the experimental setup. Therefore, the physical support in communication is realized through Ethernet.

**Figure 4:**
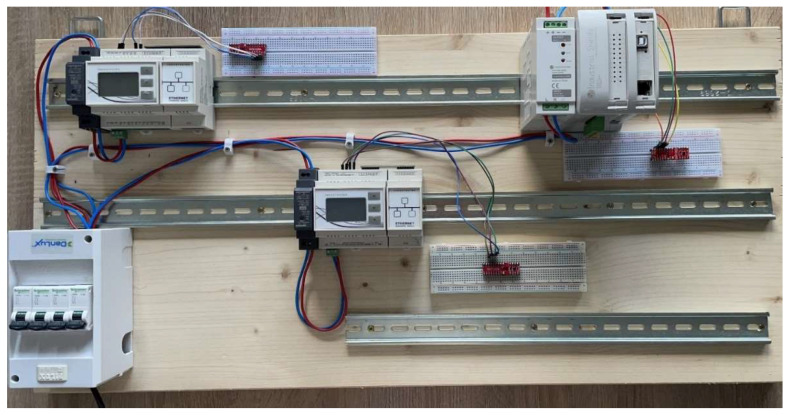
Experimental setup with three Optiga Trust X modules.

Furthermore, three Shield2Go OPTIGA TRUST X SLS 32 AIA were added as security chips to the experimental setup for implementing cryptography functions and secure key storage for each device part of the authentication cryptographic scheme. Each security chip was connected through I2C to each one of the Modbus nodes. The security chip was designed for securing IoT infrastructures, as presented in [48], where OPTIGA TRUST X SLS 32 AIA was used for securing the data exchange within a smart city light system. In order to emphasize the computational performance of the OPTIGA TRUST X SLS 32 AIA, the authors analyzed the use case where the cryptographic operations were executed by the application software running on the hardware devices used for master-slave node communication. The extended experimental setup from hardware perspective is shown in Figure 4. Since the Optiga Trust X is an additional element added for the authentication scheme, the experimental setup allows the deployment of the proposed concept without it.

In order to achieve the intended functionality of the hardware setup and to allow the evaluation of the latency introduced by each cryptographic operation, several open-source C language libraries were used, as follows:

- Arduino Optiga Trust X library for integration of Optiga Trust X [51].
- MsgModbus for Modbus TCP protocol [52].
- Modbus TCP Industrial Shields library for Modbus TCP protocol custom made for MDUINO PLC [53]
- RELIC library for cryptographic operations [54].

To integrate the Optiga Trust X software stack [55] with the Industruino and MDUINO devices, the Arduino Optiga Trust X standard open-source crypto software library was selected and configured for each device in I2C communication through the platform abstraction layer. The protocol defined by Infineon [56] allows single-master/multi-slave configuration and a clock speed configuration of up to 1 MHz, where, over the frequency of 400 KHz, the security chip will run in fast mode. Considering that the frequency set must be supported by all devices connected to the I2C bus, we chose the 400 KHz as the clock speed. Additionally, the current limitation was set to 15 mA, which allowed for the execution of functions at the highest performance.

MsgModbus was chosen due to its capability to send/receive messages on Modbus TCP, acting as either a master or as a slave. More than that, it has a scalable design following the Modbus specification, which allows integration with other C language software libraries. MsgModbus was updated by incorporating a functional application layer responsible for handling modified messages with an additional payload and the integration of cryptographic functions.

For the MDUINO device, the Modbus TCP library from Industrial Shields was used as it was custom built for this equipment, following the Modbus specifications. This library was modified in a similar manner to the MsgModbus library, since they have the same goals in deploying the system concept.

The RELIC library was selected due to its flexible configuration and its scalability in terms of building efficient custom cryptographic toolkits, allowing for the implementation of different security solutions. More than that, it supports the configuration of a variety of elliptic curves over both primary and binary fields, including the ones specified by NIST, among which are NIST P-160, NIST P-192 and NIST P-256. Additionally, the cryptographic library implements cryptographic protocols such as ECDSA, ECIES, ECDH and AES together with block cyphers, along with multiple-precision integer arithmetic, bilinear maps and related extension fields. Its performance was evaluated by [57,58,59], where it was proven that the library was more efficient in terms of duration for the analyzed cryptographic operations than other existing open-source cryptographic libraries.

### Cryptographic Library Setup

The RELIC library supports build configurations with a reduced number of cryptographic operations for various hardware architectures, including for low memory microprocessors such as ATMEGA2560. The low memory configuration uses assembly instruction, which supports the efficient execution of complex mathematical operations from a memory resources perspective. Additionally, in terms of assembly mathematics, we chose to use binary elliptic curves on a 160-bit with right-to-left width-w NAF as the point multiplication method and Interleaving of w-(T)NAF as the simultaneous point multiplication method. Therefore, for the MDUINO device, we used the low memory configuration with the options mentioned above. For the Industruino devices, which have more memory resources at their disposal, we configured and built the RELIC library without using assembly mathematics in order to allow for the selection of several primary field elliptic curves for key generation with a size higher than 160 bit, and also for deploying the ECIES-based proposed concept. Additionally, we configured the primary elliptic curve left-to-right width-w NAF as the point multiplication method and Interleaving of w-(T)NAF as the simultaneous point multiplication method. The NAF-based multiplication methods were selected due to their efficiency for hardware devices with memory constraints, as presented by [60,61].

## 5. Results and Discussion

To evaluate the proposed concept for both the authentication scheme and the hybrid encryption scheme, we measured the duration of each cryptographic operation executed by Optiga Trust X and by the devices selected for the experimental setup, which can be used in a real automation/SCADA system. In addition, we analyzed the latency introduced by the cryptographic functions necessary for an exchange of Modbus TCP messages over a secure channel in contrast with the real-time requirements of the industrial system. Furthermore, the security strength introduced by the proposed concept and the protection against cyber-attacks are analyzed and discussed. However, the security level introduced by the proposed concept is discussed first in order to allow for a better comparison of each ECC curve used. As defined by NIST in [62], the security strength given by a key is dependent on the length of the binary expansion of the primary field (i.e., key size). Therefore, with respect to the security strength of the ECC-based cryptographic algorithm, the security level introduced is driven by the strength of the security key used, as shown in Table 1, where a comparison with RSA is presented. Additionally, it can be observed that the same level of security as RSA can be achieved with smaller key sizes of ECC keys. In this context, when comparing two cryptographic algorithms from a security perspective, the security level is related to the security strength given by the key size of a symmetric algorithm, as presented by [62].

The duration of each cryptographic operation associated with the authentication scheme performed by Optiga Trust X are shown in Table 2. Considering that the key pair generation is carried out once in a third-party environment, it will not be included in the evaluation of the authentication scheme during runtime, but it will be separately addressed. Considering this aspect, it was observed that the total duration during runtime of an authentication procedure between two devices was 0.249063 s.

The measurements for ECDSA functions based on the RELIC library were conducted with the purpose of demonstrating the advantages of using a crypto processor in respect to latency and security strength. Table 3 shows that Optiga Trust X is faster, with 0.155237 s for a 256-bit key size, than the authentication scheme executed on the Industruino devices based on a 160-bit key size for ECDSA. The 192-bit increases the security strength, but it introduces a 0.2331 s latency for the signature generation and verification, and 0.06102 s for key pair generation.

Furthermore, when comparing the duration of the ECDSA functions for the signing and verifying executed by the MDUINO device for a 160-bit ECC curve, as shown in Table 4, it can be observed that the Industruino devices are faster, at 0.239696 s for the same key length, while Optiga Trust X has a 0.394933 s higher performance. Therefore, in addition to the secure key storage feature, Optiga Trust X offers higher security strength with lower computational cost than the ECDSA functions executed by both Industruino and MDUINO devices. In respect to the key pair generation, the higher duration is measured by the functions executed by the MDUINO device, which has a slower clock speed than the Industruino devices and a different Relic configuration due to low memory resources. The obtained results are supported by [63], where it was pointed out that ECDSA has a better performance for the same ECC curves on devices running at higher clock frequencies.

In respect to the hybrid encryption scheme, the measurements results from Table 5 emphasize that the NIST P-160 has a better computation performance than NIST P-192. As result, in comparison with NIST P-160, the key pair generation NIST P-192 introduces an additional computation cost of 0.05778 s, while, for the encryption and decryption procedure, the total duration is higher, at 0.35124 s. Nevertheless, in both cases, the real-time constraints are fulfilled since the total duration of deploying the proposed concept between master and slave takes 0.55094 s for the 160-bit key size, and 0.90218 s for the 192-bit key size; though this time does not include the ECC key pair generation, which is not executed during runtime.

Having a larger message for either sending or receiving messages on Modbus TCP will require an additional time on the communication network, so when choosing the key size, one must consider all characteristics, including security strength introduced versus added latency. For the authentication scheme concept based on the ECDSA algorithm, the additional size of the original message m consists of the signature S, which is double that of the key size. Therefore, for the operation executed by Optiga Trust X with an ECC key size of 256 bit, the additional size of the original transmitted Modbus TCP message will be 512 bit. If an authentication method based on the RELIC library is used instead, the additional size will be 384 bit for the 192-bit key size and 320 bit for the 160-bit key size. For the hybrid encryption scheme, the size of the output message will vary based on the key size length, as observed from Table 6, where the results are presented for the chosen ECIES configuration. Since the message m is transmitted or received encrypted, the additional size of the original Modbus TCP message is the result of the ECIES output message minus the size of the Modbus TCP message before encryption. Therefore, when considering a request message m for reading a coil with a size of 96 bit, for a key size of 192 bit, the encrypted Modbus TCP message will have a length of 576 bit, which introduces an additional size of 480 bit compared to the unencrypted Modbus TCP message.

**Figure 5:**
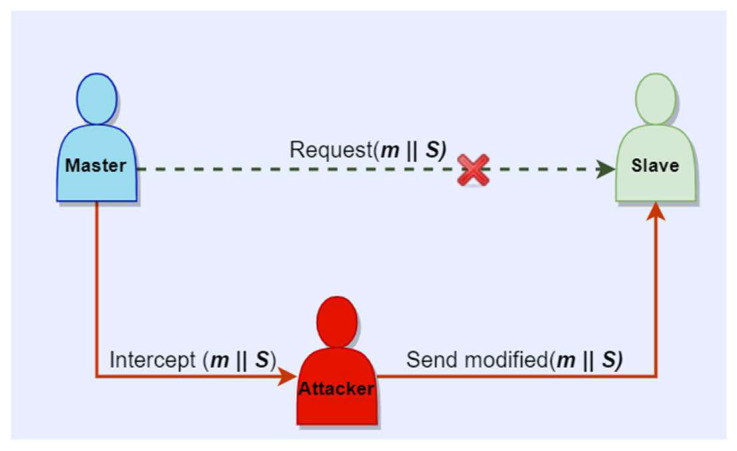
MITM scenario for authentic master-slave communication.

**Figure 6:**
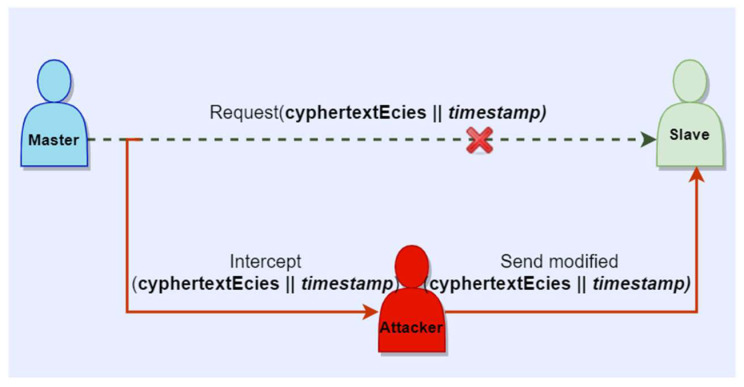
Replay attack for encrypted master-slave communication.

With respect to known cyber-attacks, we can argue that the proposed concept offers protection against MITM and replay attacks. In a scenario of communication between a master and a slave, an intruder can intercept the message m, change its content and send it to the intended party, as described in Figure 5. With respect to the authentication scheme based on ECDSA, since the attacker does not know the secret used for the signature generation S, they will not be able to re-compute a valid signature. The receiver, which in this scenario is the slave, computes the hash over the modified message and verifies the signature, which in this case will be unsuccessful since the content of the message was changed. Moreover, in the context of ECIES, if an attacker eavesdrops on the communication channel, intercepts the encrypted message cyphertextEcies and changes its content, the receiver will be able to determine that the message is not authentic by computing the HMAC over the encrypted message c, and thus mitigating malleability attacks. Furthermore, in the scenario where an attacker will replay the intercepted message unchanged, having a timestamp attached to the original message can offer protection against replay attacks, as emphasized in [64]. In the context of ECDSA, the timestamp will be concatenated to the message m and signature S. In this way, the receiver will be able to verify the freshness of the message and discard it if it does not fit with the time requirements. In an attack scenario, as described in Figure 6, in the context of ECIES, when the encrypted message concatenated with the timestamp is intercepted by an attacker, it cannot be replayed in the same time with the original message. Therefore, the receiver will analyze the replayed message and it will discard it, since the timestamp will show that the message is too old. Worth mentioning is that if the authentication procedure is executed before the encryption of the communication channel, the devices will be able to verify that the transmitted or received messages are authentic, which will provide additional security.

## 6. Conclusions

This paper presents an elliptic curve cryptography-based working solution for securing legacy communication protocols, such as Modbus TCP, with emphasis on its integration in lower-level industrial devices used within a real SCADA and automation system architecture. Additionally, it emphasizes the advantages of using Optiga Trust X as a secure cryptographic microprocessor instead of a software-based only solution, which is a hardware device designed specifically for addressing IoT requirements.

Two methods are proposed for securing the Modbus TCP communication channel between two devices to respond to the needs of assuring authenticity, integrity and confidentiality. The first method uses Optiga Trust X as a security chip for deploying the ECDSA authentication scheme. One of the main advantages of the Optiga Trust X is the secure storage of the ECC keys, which brings an advantage from a security perspective. In addition, as presented in the paper, compared to a software-based solution, the Optiga Trust X offers a much lower computational cost with a higher security level due to the capability of a fast execution of cryptographic operations. Nevertheless, the ECDSA software-based authentication scheme fulfills the time constraints requirements, but raises security concerns in respect to efficient methods of secure storing for the ECC keys. The hybrid encryption scheme ECIES encrypts the communication over the Modbus TCP channel in order to assure confidentiality and integrity for the exchanged messages. As presented, for the chosen ECC curves, the computational cost increases based on the key size, along with the security level introduced. Therefore, NIST P-192 has a higher computational cost and a higher security level than NIS P-160, but both are within the acceptable communication time for Modbus TCP. In order to obtain a secure trusted channel, the authentication procedure must first take place, and then the hybrid encryption scheme. Nevertheless, the two methods can be independently used considering the requirements of the targeted SCADA and automation system architecture. With respect to protection attacks, the analysis shows that both methods are resistant against targeted types of attacks.

The paper demonstrated, through measurements, that the proposed concept fulfills the time constraints of a real SCADA network for Modbus TCP communication while, being integrated into and deployed on Industruino and MDUINO PLCs. Additionally, it proves that security algorithms can be implemented on existing devices from the industry without the need for replacing them with new devices that have built-in security mechanisms, which is a costly and invasive action. Considering these aspects, the proposed concept has real potential to be deployed in the industry among critical infrastructures and adopted for securing other communication protocols.

## Figures and Tables

**Table 1:** Analysis of the security level for each ECC key size in comparison with RSA.

ECC Key Size (Bit)	RSA Key Size (Bit)	Security Level (Bit)
≥160 & ≤223	1024	≤80
256	3072	128

**Table 2:** Measurements of the executed Optiga Trust X functions related to the ECDSA-based authentication scheme.

Optiga Function	Duration (s)
GenerateKeyPair	0.075688
CalculateSign	0.098863
VerifySignature	0.150200

**Table 3:** Measurements of the executed ECDSA functions based on the RELIC library by the Industruino devices.

ECDSA Function	Duration (s)	
ECC Key size (bit)	160	192
GenerateKeyPair	0.099500	0.160520
CalculateSign	0.109280	0.171800
VerifySignature	0.295020	0.465600

**Table 4:** Measurements of the executed ECDSA functions based on the RELIC library on the MDUINO device.

ECDSA Function	Duration (s)
GenerateKeyPair	0.213076
CalculateSign	0.252996
VerifySignature	0.391000

**Table 5:** Measurements of the executed ECIES functions by the Industruino devices.

ECIES Function	Duration (s)	
ECC Key size (bit)	160	192
GenerateKeyPair	0.100500	0.158280
EncryptMessage	0.328600	0.533180
DecryptMessage	0.222340	0.369000

**Table 6:** Output size for the message m based on ECIES.

Key Size (Bit)	Output Size (Bit)
160	544
192	576

## Data Availability

Data is contained within the article.
